# Phagocytosis of haemozoin (malarial pigment) enhances metalloproteinase-9 activity in human adherent monocytes: Role of IL-1beta and 15-HETE

**DOI:** 10.1186/1475-2875-7-157

**Published:** 2008-08-18

**Authors:** Mauro Prato, Valentina Gallo, Giuliana Giribaldi, Paolo Arese

**Affiliations:** 1 Department of Genetics, Biology and Biochemistry, University of Torino Medical School, Torino, Italy

## Abstract

**Background:**

It has been shown previously that human monocytes fed with haemozoin (HZ) or trophozoite-parasitized RBCs displayed increased matrix metalloproteinase-9 (MMP-9) enzyme activity and protein/mRNA expression and increased TNF production, and showed higher matrix invasion ability. The present study utilized the same experimental model to analyse the effect of phagocytosis of: HZ, delipidized HZ, beta-haematin (lipid-free synthetic HZ) and trophozoites on production of IL-1beta and MMP-9 activity and expression. The second aim was to find out which component of HZ was responsible for the effects.

**Methods:**

Native HZ freshly isolated from *Plasmodium falciparum *(Palo Alto strain, Mycoplasma-free), delipidized HZ, beta-haematin (lipid-free synthetic HZ), trophozoites and control meals such as opsonized non-parasitized RBCs and inert latex particles, were fed to human monocytes. The production of IL-1beta by differently fed monocytes, in presence or absence of specific MMP-9 inhibitor or anti-hIL-1beta antibodies, was quantified in supernatants by ELISA. Expression of IL-1beta was analysed by quantitative real-time RT-PCR. MMP-9 activity and protein expression were quantified by gelatin zymography and Western blotting.

**Results:**

Monocytes fed with HZ or trophozoite-parasitized RBCs generated increased amounts of IL-1beta and enhanced enzyme activity (in cell supernatants) and protein/mRNA expression (in cell lysates) of monocyte MMP-9. The latter appears to be causally related to enhanced IL-1beta production, as enhancement of both expression and enzyme activity were abrogated by anti-hIL-1beta Abs. Upregulation of IL-1beta and MMP-9 were absent in monocytes fed with beta-haematin or delipidized HZ, indicating a role for HZ-attached or HZ-generated lipid components. 15-HETE (15(S,R)-hydroxy-6,8,11,13-eicosatetraenoic acid) a potent lipoperoxidation derivative generated by HZ from arachidonic acid via haem-catalysis was identified as one mediator possibly responsible for increase of both IL-1beta production and MMP-9 activity.

**Conclusion:**

Results indicate that specific lipoperoxide derivatives generated by HZ may play a role in modulating production of IL-1beta and MMP-9 expression and activity in HZ/trophozoite-fed human monocytes. Results may clarify aspects of cerebral malaria pathogenesis, since MMP-9, a metalloproteinase able to disrupt the basal lamina is possibly involved in generation of hallmarks of cerebral malaria, such as blood-brain barrier endothelium dysfunction, localized haemorrhages and extravasation of phagocytic cells and parasitized RBCs into brain tissues.

## Background

Phagocytosis of haemozoin (HZ, malarial pigment) or HZ-containing trophozoites alters functionality of human monocytes and macrophages. Monocyte ability to perform oxidative burst is compromised [1], bacterial killing abolished [2], antigen presentation altered [3], and ability to differentiate to functional dendritic cells disturbed [4]. Moreover, HZ-laden monocytes produce increased amounts of peroxidation products of polyunsaturated fatty acids (PUFAs) [5] and stimulate generation of several cytokines, such as TNF, IL-1beta, MIP-1alpha and MIP-1beta [6,7].

It has been shown [8] that HZ/trophozoite-fed human monocytes produced increased amounts of TNF and upregulated mRNA/protein expression and activity of matrix metalloproteinase-9 (MMP-9), a proteolytic enzyme which degrades matrix proteins [9,10] and sheds TNF and IL-1beta from cell-bound precursors [11,12]. As TNF induces the synthesis of MMP-9 [13], ingested HZ was found to generate a TNF-driven positive feedback loop enhancing production of TNF and activity of MMP-9, both blocked by a specific inhibitor of MMP-9.

Here it is shown that HZ/trophozoite-fed human monocytes generated increased amounts of IL-1beta and enhanced expression and activity of MMP-9. The latter appears to be causally related to enhanced IL-1beta production, as both expression and activation were abrogated by anti-hIL-1beta Abs. It is also shown that upregulation of IL-1beta and MMP-9 was absent in monocytes fed with beta-haematin (lipid-free synthetic HZ) or delipidized HZ, indicating a role for HZ-generated lipid components. 15-HETE (15(S,R)-hydroxy-6,8,11,13-eicosatetraenoic acid), a potent lipoperoxidation derivative generated by HZ from arachidonic acid via haem-catalysis [5] was identified as one mediator possibly responsible for increased IL-1beta production and MMP-9 activity.

## Methods

### Materials

All materials were from Sigma-Aldrich, St Louis, MO, unless otherwise stated. Cell culture media RPMI 1640, Macrophage-SFM medium, TRIzol, M-MLV, oligo-dT, sense and anti-sense primers, Platinum Taq DNA Polymerase were from Invitrogen, Carlsbad, CA; Panserin 601 monocyte medium was from PAN Biotech, Aidenbach, Germany; recombinant human (rh)IL-1beta, blocking anti-human (h)IL-1beta antibodies and Merck's inhibitor I, (N-hydroxy-1-(4-methoxyphenyl)sulfonyl-4-(4-biphenylcarbonyl)piperazine-2-carboxamide), a specific inhibitor of MMP-9/MMP-13 activity, were from Merck, Darmstadt, Germany; ELISA kit for IL-1beta assay and 15-HETE were from Cayman, Ann Arbor, MI; anti-D IgG were from Immuno AG, Vienna, Austria; Percoll was from Pharmacia, Uppsala, Sweden; Dynabeads M-450 CD2 Pan T and M-450 CD19 Pan B were from Dynal, Oslo, Norway; Diff-Quik parasite stain was from Baxter Dade AG, Dudingen, Switzerland; sterile plastics were from Costar, Cambridge, UK; bicinchoninic acid protein assay was from Pierce, Rockford, IL; anti-MMP-9 monoclonal antibodies were from Santa Cruz Biotechnology, Santa Cruz, CA; DNA-free kit was from Ambion, Austin, TX; Beacon Designer 2.1 software was from Premier Biosoft International, Palo Alto, CA; dNTPs were from Applied Biosystem, Foster City, CA. 4-hydroxynonenal (HNE) was from Biomol, Plymouth Meeting, PA. Beta-haematin (synthetic HZ) was prepared according to the Slater *et al *[14] procedure, modified as indicated [15].

### Cultivation of *Plasmodium falciparum *and isolation of trophozoite-parasitized RBCs and native or delipidized HZ

*Plasmodium falciparum *parasites (Palo Alto strain, Mycoplasma-free) were kept in culture as described [4]. HZ and trophozoite-parasitized RBCs (trophozoites) isolated from cultures during the first two days after infection of RBCs were added to schizonts (multinucleated parasite form). After centrifugation at 5,000 *g *on a discontinuous Percoll-mannitol density gradient, native HZ was collected from the 0–40% interphase and trophozoites/schizonts from the 40–80% interphase [4]. Native HZ was washed five times with 10 mM HEPES (pH 8.0) containing 10 mM mannitol at 4°C and once with PBS, and stored at 20% (vol/vol) in PBS at -20°C. For delipidized haemozoin, lipid extraction was performed as previously reported [5]. After isolation, HZ and trophozoites enriched to 95–97% parasitaemia were washed twice and reincubated in RPMI 1640 for 1 h at 37°C before opsonization and phagocytosis.

### Preparation and handling of monocytes

Human monocytes were separated by Ficoll centrifugation from freshly collected buffy coats discarded from blood donations by healthy adult donors of both sexes provided by the local blood bank (AVIS, Associazione Volontari Italiani Sangue, Torino, Italy) [1]. Separated lymphomonocytes were resuspended in RPMI 1640 medium and plated on wells of six-well plates. Each well received 2 ml of cell suspension containing 8 × 10^6 ^cells/ml in RPMI 1640. The plates were incubated in a humidified CO_2_/air-incubator at 37°C for 60 min. Thereafter, non-adherent cells were removed by three washes with RPMI 1640 and adherent cells reincubated at 37°C overnight in RPMI 1640. Shortly before starting phagocytosis, wells were washed with RPMI 1640 and Macrophage-SFM medium added (2 ml/well). Adherent cells prepared by this method were detached from the plates by scraping, stained with specific antibodies and analysed on a FACScan flow cytometer (Becton-Dickinson, San Jose, CA). As an average, monocytes (CD14^+ ^cells) were 63.8 ± 5.7%, lymphocytes 36.2 ± 5.7% (mean values ± SD, n = 6) of all mononuclear cells. For selected experiments, lymphomonocytes were separated by Ficoll centrifugation from fresh buffy coats (see above) and monocytes immunopurified by depletion of non-monocytic cells from lymphomonocytes. Dynabeads M-450 CD2 Pan T and M-450 CD19 Pan B (Dynal) were added to the lymphomonocytes in a 2:1 ratio for 20 min at 4°C. B and T lymphocytes were removed by biomagnetic separation as specified by the manufacturer. The remaining monocytes were washed twice and resuspended in Macrophage-SFM medium. By this method monocytes (CD14^+ ^cells) were 73.6 ± 9.5% pure, (mean values ± SD, n = 6, range 62–89.2%).

### Phagocytosis by adherent monocytes of opsonized trophozoites, native or delipidized HZ, beta-haematin, nonparasitized opsonized RBCs and latex particles

To each well of a six-well plate with approx. 1 × 10^6 ^adherent monocytes, 50 μl trophozoites (10% haematocrit), native or delipidized HZ (120 nmoles HZ haem, an amount comparable to 50 μl trophozoites on haem content basis), 50 μl beta-haematin (120 nmoles haem), 50 μl anti-D IgG-opsonized RBCs (10% haematocrit) and 50 μl amine-modified, red-fluorescent latex particles (2.5% solids, diameter 0.105 μm) were added. Trophozoites, native and delipidized HZ, beta-haematin and latex particles were opsonized with fresh autologous serum, and nonparasitized RBCs were opsonized with anti-D IgG as indicated [1,5]. After opsonization, all phagocytic meals were suspended in Macrophage-SFM medium. The plates were centrifuged at low speed for 5 seconds to start phagocytosis and incubated in a humidified CO_2_/air-incubator at 37°C for 3 hours. This time period maximized phagocytosis and was not sufficient to induce haem-oxygenase-mediated degradation of ingested haem [16]. Thereafter, non-ingested cells, HZ, latex and beta-haematin particles were removed by four washes with RPMI 1640. The plates were then incubated in a humidified CO_2_/air-incubator at 37°C for the indicated times. In selected experiments, cells were incubated with rhIL-1beta (20 ng/ml), blocking anti-hIL-1beta antibodies (30 ng/ml) or Merck's inhibitor I, a specific inhibitor of MMP-9/MMP-13 activity (4 ng/ml) for 48 h.

### Assay of IL-1beta production

After termination of phagocytosis, monocytes were further incubated with Panserin 601 monocyte medium in a humidified CO_2_/air-incubator at 37°C for 48 h in presence (4 ng/ml) or absence of Merck's inhibitor I, a specific inhibitor of MMP-9/MMP-13 activity. The level of active soluble IL-1beta was assayed in monocyte supernatants by ELISA. A standard calibration curve was generated with rhIL-1beta, according to the manufacturer's instructions.

### Assay of MMP-9 activity by gelatin zymography

After termination of phagocytosis, monocytes were further incubated with Panserin 601 monocyte medium in a humidified CO_2_/air-incubator at 37°C for 48 h. Thereafter, the activity of MMP-9 was evaluated by gelatin zymography in the cell supernatants as indicated [8,17,18]. Supernatants were loaded on 8% polyacrylamide gels containing 0.1% gelatin under non-denaturing and non-reducing conditions. Following electrophoresis, gels were washed and incubated for 18 h at 37°C in a collagenase buffer. Densitometric analysis of the bands considered to reflect total enzymatic activity of MMP-9, was performed using a computerized densitometer (Chemidoc, Biorad, Hercules, CA) with activity presented in relative units compared to background.

### Assay of MMP-9 protein expression by western blotting

After termination of phagocytosis, monocytes were further incubated with Panserin 601 monocyte medium in a humidified CO_2_/air-incubator at 37°C for 48 h. Thereafter, cells were washed and lysed at 4°C in lysis buffer containing (mM): NaCl, 300; Tris, 50; 1% (vol/vol) Triton-X100; protease and phosphatase inhibitors: pepstatin, 50 ng/ml; leupeptin, 50 ng/ml; aprotinin, 10 μg/ml. The protein content in the lysate was measured by the bicinchoninic acid assay and 12 μg protein/lane were added to the loading buffer. The lysates samples were loaded on 8% polyacrylamide gels under denaturing and reducing conditions, with addition of Laemmli buffer, blotted on a polyvinylidene difluoride membrane, and probed with anti-MMP-9 monoclonal antibodies at 1/1,000 final dilution. Bands were visualized by enhanced chemiluminescence. Densitometric analysis of the bands was performed using a computerized densitometer (Chemidoc).

### Assay of IL-1beta mRNA expression by real-time quantitative RT-PCR

After termination of phagocytosis, monocytes were further incubated with Panserin 601 monocyte medium in a humidified CO_2_/air-incubator at 37°C for 6 h (immunopurified monocytes) or 15 h (adherent monocytes). Total cellular RNA from 2 × 10^6 ^cells was isolated from monocytes by TRIzol, according to the manufacturer's instructions, and eluted in 20 μl diethyl pyrocarbonate water. To remove any contaminating DNA, RNA was treated with Ambion's DNA-free kit (Ambion). Retrotranscription was performed using 6 μg of RNA, 200 U/μl of M-MLV and 25 μ/μl oligo-dT (Invitrogen). Real-time quantitative RT-PCR was performed with the i*Cycler *instrument (Bio-Rad) and data analysis was performed with iCycler iQ Real-Time Detection System Software version 3.0 (Bio-Rad). IL-1beta (GenBank accession no. NM_000576) oligonucleotide sequences (forward: 5'-ACA GAT GAA GTG CTC CTT CCA-3', reverse: 5'-GTC GGA GAT TCG TAG CTG GAT-3') were identified using Beacon Designer Software package and designed to be intron-spanning allowing the differentiation between cDNA and DNA-derived PCR products. PCR amplification was carried out in 25 μl of reaction mixture. 1 μl of cDNA (corresponding to 10^5 ^cells) and 400 nM primers were added to the amplification mixture (iQ SYBR Green Supermix, Bio-Rad). DNA polymerase was pre-activated for 2 min at 94°C, and the amplification was performed by a 40-cycle PCR (94°C, 30 s, 60°C, 30 s and 72°C, 30 s). Glyceraldehyde-3-phosphate dehydrogenase (GAPDH) was used as reference gene to normalize cDNA across samples. Relative quantitation for IL-1 beta, expressed as -fold variation over untreated control cells, was calculated using the 2^-ΔΔCT ^method. To validate the use of the 2^-ΔΔCT ^method, serial dilutions of cDNA from monocytes stimulated for 6 or 15 h by 20 ng/ml rhTNF, were tested. Analysed transcripts exhibited high linearity amplification plots (r > 0.98) and similar PCR efficiency (85.7% for IL-1 beta and 86.5% for GAPDH), confirming that the expression of each gene could be directly compared. The specificity of PCRs was confirmed by melt curve analysis. Values are means of triplicate measurements.

## Results

### Enhancement of IL-1beta production by adherent monocytes fed with HZ or trophozoites

**Figure 1:**
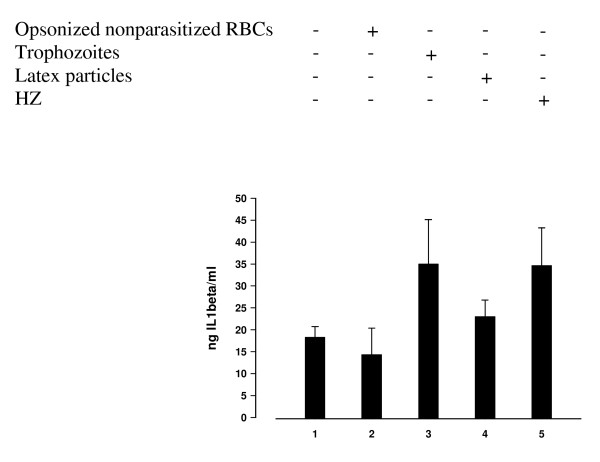
**HZ and trophozoite phagocytosis enhances IL1-beta production by human adherent monocytes**. Human adherent monocytes were unfed or fed with HZ, trophozoites and control meals (IgG-anti D-opsonized nonparasitized RBCs, latex particles). After 3 h phagocytosis and a further incubation during 48 h, IL1-beta levels were measured by ELISA in cell supernatants. Data are given as ng IL-1beta/ml supernatant (mean values ± SD of six independent experiments). Data were analysed for significance by Student's t-test. Significance of differences (column numbers): trophozoite-fed(3)/HZ-fed(5) vs unfed-(1)/nonparasitized RBC-fed(2) monocytes, p < 0.05; latex-fed(4) vs unfed-(1)/nonparasitized RBC-fed(2) monocytes, n.s.; trophozoite-fed(3) vs HZ-fed(5) monocytes, n.s.

Adherent monocytes were allowed to phagocytose HZ, trophozoites, nonparasitized opsonized RBCs and latex particles (control meals) during 3 h. As an average, each monocyte ingested 8–10 trophozoites, or HZ equivalent to 8–10 trophozoites in terms of ingested haem, or almost 8–10 non-parasitized anti-D IgG opsonized RBCs, as shown previously [1,19]. After termination of phagocytosis and elimination of noningested phagocytic meals by repeated washings, and further incubation during 48 h, IL-1beta production was measured by ELISA in cell supernatants. Compared to unfed control monocytes, IL-1beta production was increased approximately two-fold after phagocytosis of HZ or trophozoites, whereas phagocytosis of control meals did not affect significantly cytokine production (Figure 1). In selected experiments, adherent monocytes after the 3 h phagocytic period were further incubated for 48 h in presence of Merck's inhibitor I, a specific inhibitor of MMP-9/MMP-13 activity. Supplementation of this inhibitor did not affect cytokine production by HZ/trophozoite-fed monocytes.

### Enhancement of MMP-9 protein expression and enzyme activity in adherent monocytes after HZ phagocytosis and rhIL-1beta treatment. Abrogation of the HZ effect by anti-hIL-1beta antibodies

**Figure 2:**
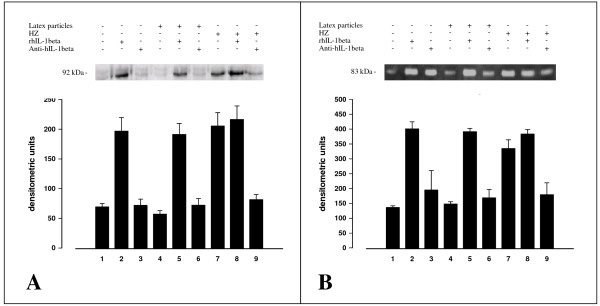
**HZ and trophozoite phagocytosis, and rhIL-1beta enhance MMP-9 protein expression (in cell lysates) and enzyme activity (in cell supernatants) in human adherent monocytes. Abrogation of the HZ effect by anti-hIL-1beta antibodies**. Human adherent monocytes were unfed, fed with HZ or latex particles treated or not with rhIL-1beta (20 ng/ml) or blocking anti-hIL-1beta antibodies (30 ng/ml) as indicated. **Panel A**. Western blot with anti-MMP-9 antibodies and densitometric quantification of MMP-9 protein. After 3 h phagocytosis and a further incubation during 48 h, cell lysates were prepared, separated by PAGE (8% polyacrylamide) blotted and probed with anti-MMP-9 monoclonal antibodies (1/1000 final dilution). The 92-kDa band in the gel corresponds to pro-MMP-9. Data are given as arbitrary densitometric units (mean values ± SD of four independent experiments). **Panel B**. Gelatin zymography and densitometric quantification of MMP-9 enzyme activity. After 3 h phagocytosis and a further incubation during 48 h, cell supernatants were separated by PAGE (8% polyacrylamide gel containing 0.1% gelatin) under non-denaturing and non-reducing conditions. The 83-kDa negative bands in the gel correspond to MMP-9 enzyme activity. Data are given as arbitrary densitometric units (mean values ± SD of four independent experiments). Data (Panel A, Panel B) were analysed for significance by Student's t-test. Significance of differences (column/lane numbers): HZ-fed(7)/rhIL1beta(2)-stimulated vs control(1)/anti-hIL1beta-stimulated(3)/latex-fed(4) monocytes, p < 0.01 (Panel A) or p < 0.05 (Panel B). rhIL1beta-stimulated(2,5,8) vs HZ-fed(7,8) monocytes, n.s.; anti-hIL1beta-stimulated(3,6,9)/latex-fed(4) vs unfed(1) monocytes, n.s.

Unfed, latex-fed and HZ-fed adherent monocytes after termination of phagocytosis were further incubated for 15 h (MMP-9 mRNA expression studies) or 48 h (MMP-9 activity and protein expression studies) in presence or absence of 20 ng/ml rhIL-1beta and 30 ng/ml blocking anti-hIL-1beta antibodies. Phagocytosis of HZ enhanced enzyme activity (measured in cell supernatants) and protein expression (measured in cell lysates), confirming previous data obtained by this group [8] (Figure 2, panel A). rhIL-1beta added to unfed or latex-fed monocytes mimicked the HZ effect at the protein expression/enzyme activity level (Figure 2, panel A and panel B) and at the mRNA expression level, and further enhanced enzyme activity when added to HZ-fed monocytes (Figure 2, panel A). Blocking anti-hIL-1beta antibodies abrogated the enhancement of protein expression (Figure 2, panel A) and enzyme activity (Figure 2, panel B), and also inhibited enhancement of mRNA expression observed after HZ phagocytosis.

### Role of lipidic component of HZ in enhancement of IL-1beta production and MMP-9 activity in adherent monocytes after phagocytosis of HZ

**Figure 3:**
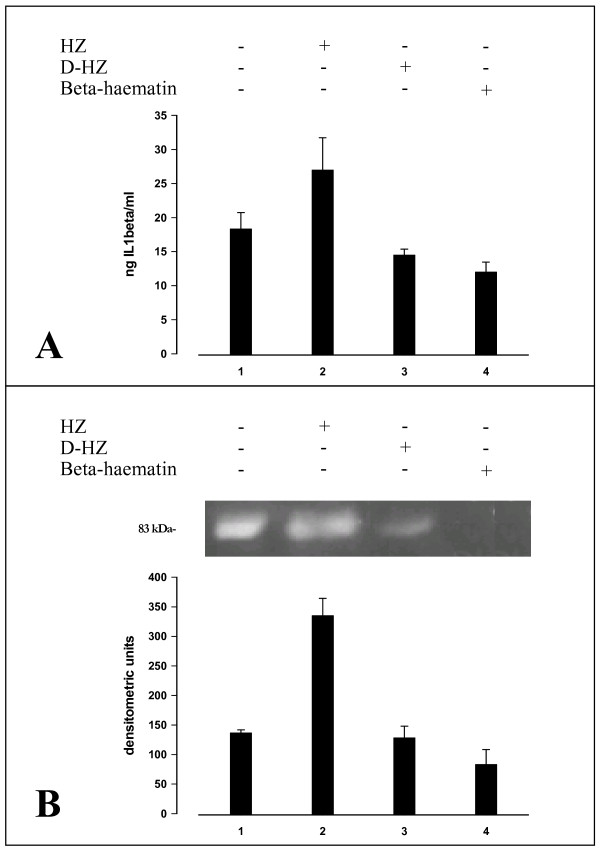
**IL-1beta production and MMP-9 enzyme activity (in cell supernatants) in human adherent monocytes unfed or fed with HZ, delipidized HZ or beta-haematin**. Human adherent monocytes were unfed or fed with HZ, delipidized HZ (D-HZ) and beta-haematin. **Panel A: **IL-1beta production. After 3 h phagocytosis and a further incubation during 48 h, IL1-beta levels were measured by ELISA in cell supernatants. Data are given as ng IL-1beta/ml supernatant (mean values ± SD of four independent experiments). Data were analysed for significance by Student's t-test and differences between delipidized HZ or beta-haematin against unfed controls were not significant. **Panel B: **Gelatin zymography and densitometric quantification of MMP-9 enzyme activity. After 3 h phagocytosis and a further incubation during 48 h, cell supernatants were separated by PAGE and MMP-9 enzyme activity measured by gelatin zymography and densitometric quantification (see legend to Figure 2 for details). The 83-kDa negative band in the gel corresponds to MMP-9 enzyme activity. Data are given as arbitrary densitometric units (mean values ± SD of four independent experiments). Data (Panel A, panel B) were analysed for significance by Student's t-test. Significance of differences (column/lane numbers). HZ-fed(2) vs unfed(1)/D-HZ-(3)/beta-haematin(4)-fed monocytes, p < 0.01; unfed(1) vs D-HZ(2)/beta-haematin(4)-fed monocytes, n.s.

Previous work has shown that PUFAs stably adherent to the crystalline poly-haem core of native HZ are transformed by non-enzymatic haem catalysis into a number of potent lipoperoxidation derivatives [5]. To ascertain whether lipids were involved in HZ-elicited activation of MMP-9, lipid-free beta-haematin (synthetic HZ) and delipidized native HZ were fed to adherent monocytes. After phagocytosis, monocytes were further incubated for 48 hours and cell supernatants analysed by ELISA for IL-1beta production and MMP-9 activity. Beta-haematin and delipidized HZ were unable to enhance IL-1beta production (Figure 3, panel A) and stimulate MMP-9 activity (Figure 3, panel B).

### Involvement of 15-HETE in HZ-mediated effects on IL-1beta production and MMP-9 activity in adherent monocytes and on IL-1beta mRNA expression in immunopurified monocytes

**Figure 4:**
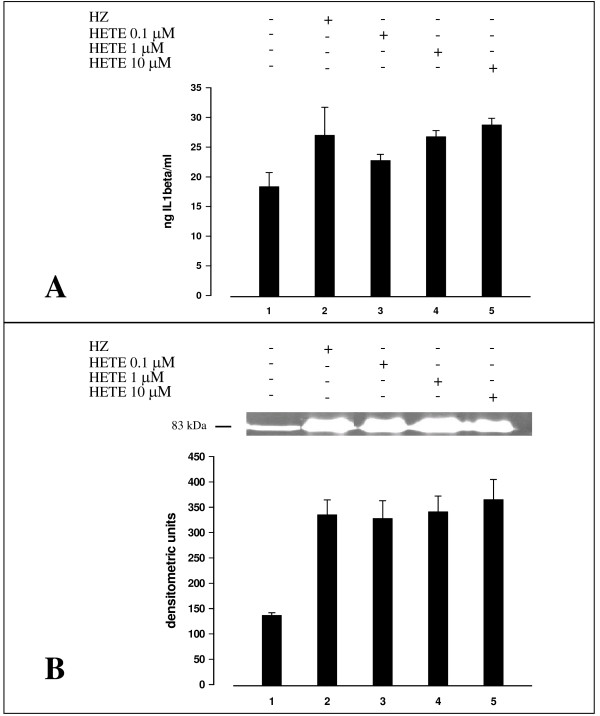
**IL-1beta production and MMP-9 enzyme activity (in cell supernatants) in human adherent monocytes unfed or fed with HZ and treated or not with 15-HETE**. Human adherent monocytes were fed or not with HZ and treated or not with 15-HETE added at time 0 at 0.1–10 μM (final concentration). **Panel A**. After 3 h phagocytosis and a further incubation during 48 h (HZ-fed monocytes) or 48 h after addition of 15-HETE, IL-1beta levels were measured by ELISA in cell supernatants. Data are given as ng IL-1beta/ml supernatant (mean values ± SD of four independent experiments). **Panel B**. After 3 h phagocytosis and a further incubation during 48 h (HZ-fed monocytes) or 48 h after addition of 15-HETE, cell supernatants were separated by PAGE and MMP-9 enzyme activity measured by gelatin zymography and densitometric quantification (see legend to Figure 2 for details). The 83-kDa negative bands in the gel correspond to MMP-9 enzyme activity. Data are given as arbitrary densitometric units (mean values ± SD of four independent experiments). Data were analysed for significance by Student's t-test. Significance of differences (column/lane numbers). Unfed(1) vs HZ-fed(5) monocytes, p < 0.05; unfed(1) vs 15-HETE-treated (3,4,5) monocytes, p < 0.05 (Panel A) and p < 0.01 (Panel B).

**Figure 5:**
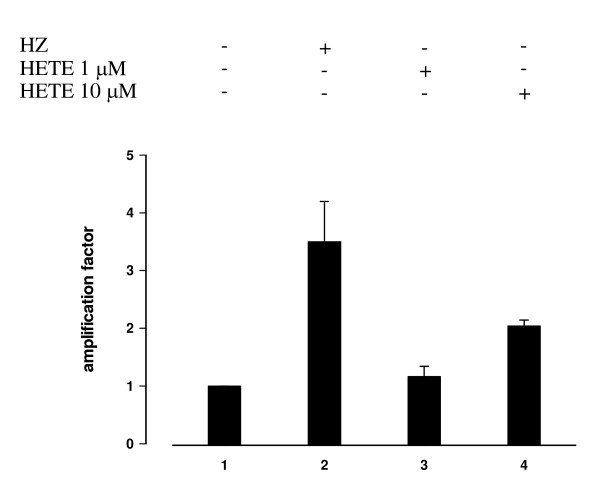
**IL-1beta mRNA expression in immunopurified human adherent monocytes unfed or fed with HZ and treated or not with 15-HETE**. Human CD14^+ ^immunopurified monocytes were unfed, fed with HZ or treated with 15-HETE added at time 0 at 1–10 μM (final concentration). 6 h after phagocytosis or addition of 15-HETE. mRNA expression was measured by real-time quantitative RT-PCR in cell lysates and expressed as -fold variation over untreated monocytes. Mean values ± SD of three independent experiments. Data were analysed for significance by Student's t-test. Significance of differences (column numbers). Unfed(1) vs HZ-fed(2) monocytes, p < 0.01; unfed(1) vs 15-HETE 1 μM(3) treated monocytes, n.s.; unfed(1) vs 15-HETE 10 μM(4) treated monocytes, p < 0.05.

Previous work has indicated that 15-HETE, a product of arachidonic acid peroxidation by HZ, is an active mediator of HZ/trophozoite effects in monocytes [20]. As shown in Figure 4, panel A, 15-HETE added to adherent monocytes at 0.1–10 μM (final concentration) enhanced production of IL-1beta, measured in cell supernatants 48 h after addition. 15-HETE added in the same concentration range also stimulated MMP-9 activity, similarly measured in cell supernatants 48 h after addition (Figure 4, panel B). This second effect was not concentration-dependent. Both 15-HETE-mediated effects were comparable to those elicited by HZ/trophozoite phagocytosis. In selected experiments with immunopurified monocytes, IL-1beta mRNA expression measured 6 h after phagocytosis was increased 3,5-fold after HZ and 2-fold after addition of 10 μM 15-HETE (Figure 5). The stimulatory effect of 15-HETE appears to be specific, as 4-hydroxynonenal [4-HNE], another potent PUFA derivative generated by HZ activity [21] was unable to stimulate IL-beta production and MMP-9 activity when added at 0.1 μM (final concentration) and downregulated both parameters when added at 1–10 μM (final concentration).

## Discussion

Matrix metalloproteinases (MMPs) are a family of zinc-dependent enzymes characterized by their ability to remodel/disrupt subendothelial matrix proteins and shed or activate cytokines from their precursors [9-12,22]. MMPs play physiological roles, for example in wound repair [22], and are also involved in pathological processes such as cancer metastasis [23] or neurological diseases [24]. Basal transcription level of MMPs is generally low [25], but it can be enhanced in various cell types including monocytes, by cytokines and growth factors, and by cell-cell or cell-matrix interactions [9]. Recently, involvement of MMPs in malaria has been described. Deininger *et al *[26] found higher levels of MMP-1 and angiogenic proteins such as VEGF in post-mortem samples of brain tissues of patients dead from cerebral malaria. Van den Steen *et al *[27] described higher MMP-9 expression in brain and other tissues of mice with cerebral malaria. The enzyme was apparently produced by cells of monocytic lineage. Lastly, present group [8] has shown that human adherent monocytes fed with HZ or HZ-containing trophozoites displayed increased activity and mRNA/protein expression of MMP-9, and increased production of TNF. Since TNF induces expression of MMP-9, while MMP-9 sheds TNF from its membrane-bound precursor, interaction between MMP-9 and TNF was considered to start a positive feedback loop eventually enhancing the pathological effects of both molecules: for MMP-9 – disruption of subendothelial basal lamina and infiltration of mononuclear cells in brain, lung and kidney – [28-30]; and for TNF, – fever, hypoglycaemia, circulatory failure, and placental pathology – [31-35].

Present data show that IL-1beta production was enhanced in HZ/trophozoite-fed adherent monocytes, and causally related to enhancement of MMP-9 mRNA and protein expression (measured in cell lysates) and MMP-9 enzyme activity (measured in cell supernatants). Short-term experiments were performed to establish which cytokine (TNF or IL-1beta) was the primary target of HZ stimulatory activity. Data indicate that while blocking anti-hIL-1beta antibodies significantly reduced TNF production by HZ-fed cells at 1 h (p < 0.05) and at 2 h (p < 0.02), blocking anti-hTNF Abs did not affect the short-term production of IL-1beta by HZ-fed cells. These data seem thus to suggest that the enhancement of IL-1beta formation occurred first followed by enhanced formation and activity of TNF and MMP-9, respectively.

HZ and HZ-containing trophozoites contain large amounts of monohydroxy derivatives of polyunsaturated fatty acids (OH-PUFAs). OH-PUFAs are stable derivatives of PUFA peroxidation, here most likely non-enzymatically generated by haem-catalyzed lipid peroxidation carried out by the poly-haem moiety of HZ [5]. High concentration of HZ and acidic conditions are likely to favour unspecific haem-catalyzed lipid peroxidation, leading to a complex pattern of oxygenated products. Six HETE isomers and two major isomers of HODE (hydroxyoctadeca-9Z,11E-dienoic acid, a linoleic acid derivative) were found in HZ and trophozoites [5]. Of those molecules and isomers, only 12- and 15-HETE mimicked toxic effects of HZ/trophozoite phagocytosis in monocytes, such as inhibition of oxidative burst and inhibition of differentiation and maturation of monocytes to dendritic cells [15]. HODE isomers were inactive. Native HZ was found to contain 0.24 mmole 15-HETE/mole haem. 15-HETE is further produced by ingested HZ, and HZ-fed monocytes were found to shed 15-HETE into the supernatant and to contain approximately 10 μM 15-HETE, under the realistic assumption of 10 RBC equivalents per monocyte and a monocyte volume of 500 fL [5]. 4-HNE, another PUFA derivative [21], supplemented here at 0.1 μM concentration did not stimulate IL-beta production or MMP-9 activity, while it downregulated both parameters at 1–10 μM concentration. Possible explanations may reside in the strong reactivity of 4-HNE with thiols, such as reduced glutathione, or amino-groups present in suspending buffers. Additionally, 4-HNE tends to concentrate in the cell membrane where it generates adducts with His and Cys residues of membrane proteins, possibly interfering with the 15-HETE transduction pathway.

Based on present data, following sequence of events is likely. First, 15-HETE, possibly together with co-generated, similarly active 12-HETE, would induce production of IL-1beta and TNF via a yet undetermined transduction pathway. It is likely that the NF-kB pathway is involved in cytokine upregulation, as Jaramillo *et al *recently reported activation of the NF-κB pathway in HZ-fed murine macrophages [36,37]. Subsequently, increased IL-1beta and TNF would upregulate MMP-9 expression and activity. Indeed, literature data indicate the NF-KB pathway as essential for both TNF and IL-1beta induction, and the latter cytokines as potent upregulators of MMP-9 [38,39].

## Conclusion

Phagocytosis of HZ or trophozoite-parasitized RBCs was shown to induce enhanced production of TNF and IL-beta, and to increase mRNA and protein expression (both measured in cell lysates), and enzymatic activity of MMP-9 (measured in cell supernatants), a metalloproteinase involved in disruption of basal membranes. Present data indicate that lipid components attached to HZ were instrumental for enhanced production of IL-1beta and MMP-9 increase. In fact, the ability of HZ to non-enzymatically generate HETEs and the presence of HETEs in HZ-fed monocytes [5], the lack of effects by feeding cells with delipidated HZ, and the recapitulation of HZ effects by supplementing exogeneous 15-HETE are converging indications that 15-HETE, an active member of the HETE family, may be causally involved in upregulation of IL1-beta and MMP-9. Thus, HZ-derived 15-HETE might be a molecule primarily responsible for cytokine induction and MMPs activation and possibly instrumental in inducing hallmarks of cerebral malaria such as localized haemorrhages and extravasation, migration and perivascular accumulation of phagocytic cells. Interestingly, it was recently shown that soluble factors released by trophozoite-parasitized RBCs significantly decreased electrical resistance of human brain-blood barrier endothelium, indicating a parasite-mediated perturbation of the brain monolayer barrier function [40].

## Authors' contributions

MP designed the research, performed the experiments and drafted the manuscript. VG performed the experiments and helped to draft the manuscript. GG helped with the real-time quantitative RT-PCR experiments. PA helped design the research, examined and interpreted the data and wrote the final manuscript. All authors read and approved the final manuscript.
